# Chronic Kidney Disease Patients Visiting Various Hospital Departments: An Analysis in a Hospital in Central Tokyo, Japan

**DOI:** 10.3390/jpm12010039

**Published:** 2022-01-04

**Authors:** Akira Fukui, Kohei Takeshita, Akio Nakashima, Yukio Maruyama, Takashi Yokoo

**Affiliations:** 1 Division of Nephrology and Hypertension, Department of Internal Medicine, The Jikei University School of Medicine, Tokyo 105-8461, Japan; sakn21208@hotmail.com (A.N.); maruyama@td5.so-net.ne.jp (Y.M.); tyokoo@jikei.ac.jp (T.Y.); 2 Department of Innovation for Medical Information Technology, The Jikei University School of Medicine, Tokyo 105-8461, Japan; k.takeshita@jikei.ac.jp

**Keywords:** CKD, proteinuria, dipstick test, albuminuria, eGFR, clinical departments, electronic medical records, nephrologist referral, quality indicator, early intervention

## Abstract

To further improve care for chronic kidney disease (CKD) patients, healthcare providers’ awareness of CKD must be raised. Proteinuria testing is essential for CKD care, and collaboration with specialists is recommended for advanced cases. We reviewed data from the electronic medical records of outpatients at our hospital to analyze the clinical departments visited by CKD patients, and the frequency of proteinuria testing and referrals to nephrologists. We defined CKD as an estimated glomerular filtration rate (eGFR) < 60 mL/min/1.73 m^2^ or a urine protein concentration (U-pro) ≥ +1. We found that 31.1% of the CKD tests in September 2021 were performed in clinical departments other than internal medicine. Furthermore, within 1 year, 68.0% of CKD patients identified in September 2020 underwent a urine dipstick test, and 33.7% underwent a quantitative test for urinary protein or albumin. Additionally, 27.5% of individuals with an eGFR < 30 mL/min/1.73 m^2^ or U-pro ≥ +1 identified by non-nephrology departments in September 2020 visited the nephrology department within 1 year. Repeated assessments of these quality indicators may be useful for progress management in improving CKD care. Because CKD patients visited various departments in our hospital, campaigns to raise CKD awareness must reach a wide range of healthcare providers in hospitals.

## 1. Introduction

Chronic kidney disease (CKD) has a major impact on global health, and a reduction in its burden deserves greater attention [1,2]. In 2008, the Japanese government started implementing measures against CKD centered on raising public awareness, and increasing early intervention through collaboration between primary care physicians and nephrologists. This contributed to a decrease in the age-adjusted incidence rates of dialysis [3], combined with the effects of preventive measures against lifestyle-related diseases [4], several health check-up programs [5], and recent treatment advancements. However, due to the increase in the elderly population, the annual number of new dialysis patients has not decreased. Therefore, in 2018, the Japanese government revised CKD measures, such as the use of criteria for referral to nephrologists, and set a new goal of reducing the annual number of new dialysis patients by 10% within 10 years [6]. We believe that new strategies need to be developed to reach this lofty goal because the elderly population will continue to increase.

Improving CKD care requires raising CKD awareness for both patients and healthcare providers [7,8,9]. Many CKD patients and individuals at high risk of developing CKD, including those with diabetes, hypertension, cardiovascular disease (CVD), cancer, and nonalcoholic fatty liver disease [10,11,12,13,14], visit general hospitals. There are many opportunities to screen CKD, such as screening, routine follow-up, and preoperative examinations in various hospital departments. Furthermore, because general hospitals have clinical departments, such as nephrology, diabetes, and cardiology, it is considered easier to collaborate with specialists to care for CKD patients. Thus, raising CKD awareness among healthcare providers in general hospitals, and strengthening in-hospital medical collaboration [15,16,17,18,19] may be effective strategies to improve CKD care.

We hypothesized that in-hospital CKD awareness campaigns and progress management using quality indicators (QIs) that can be expected regardless of the clinical department could be new strategies for improving CKD care. Therefore, in this study, we first determined the scope of potential awareness campaigns by analyzing the clinical departments visited by CKD patients. This was done by reviewing the electronic medical record data of outpatients at our hospital using the data warehouse CLISTA! [20]. Then, we analyzed the frequency of testing for proteinuria or albuminuria, which constitutes the definition of CKD, because these are robust and early predictors of high-risk individuals, and an important target for effective intervention [21,22,23,24,25,26]. Lastly, we analyzed the frequency of referral to a nephrologist compared with that recommended for advanced CKD [6,22,27,28,29] (pp. 112–119, [21]).

## 2. Materials and Methods

We conducted a retrospective cohort study. All data were extracted from the electronic medical records of outpatients at Jikei University Hospital using the data warehouse CLISTA! 3.5 (Medical Engineering Institute, Inc., Tsu, Japan). The estimated glomerular filtration rate (eGFR) was automatically calculated and reported using the GFR equation designed for the Japanese population, as follows: eGFR = 194 × (sCr)^−1.094^ × (age)^−0.287^ (×0.739, if female), where sCr = serum creatinine (mg/dL) [30]. Urine protein concentration (U-pro) was determined using a urine test strip (Uriflet S 9UB; ARKRAY, Inc., Kyoto, Japan), and categorized as negative, ± (10 mg/dL), +1 (30 mg/dL), +2 (100 mg/dL), +3 (300 mg/dL), or +4 (>600 mg/dL). We defined CKD as an eGFR <60 mL/min/1.73 m^2^ or a U-pro result ≥ +1 at least once during the target period. Individuals undergoing renal replacement therapy were included. Individuals aged <18 years were excluded.

### 2.1. Clinical Departments Visited by CKD Patients

We analyzed the number of clinical departments that performed each measurement where the eGFR was <60 mL/min/1.73 m^2^ or U-pro was ≥+1 at least once during September 2021. We identified CKD separately with eGFR < 60, or with eGFR ≥ 60 and U-pro ≥ +1. For the U-pro data, only those measured on the same day as the eGFR were used and counted together as one measurement. When the departments that performed eGFR and U-pro were different, the department that performed the U-pro was analyzed because U-pro is essential to identify CKD in this case.

### 2.2. Status of Care for CKD Individuals

The status of CKD care for all CKD patients identified by an eGFR < 60 mL/min/1.73 m^2^ or a U-pro test ≥ +1 at least once in September 2020 was analyzed within 1 year after identification.

#### 2.2.1. Frequency of Testing for Proteinuria or Albuminuria

We analyzed the number of CKD patients who underwent tests for proteinuria, including qualitative urine protein tests (dipstick tests) and quantitative urine protein tests of the urine protein-to-creatinine ratio (PCR) and the urine albumin-to-creatinine ratio (ACR), at least once from September 2020 to 30 September 2021.

#### 2.2.2. Frequency of Referral to Nephrologists

We analyzed the number of CKD patients who were identified according to the results of tests performed from 1 September 2020 to 30 September 2020 by non-nephrology departments. We then determined the number of these individuals who visited the nephrology department at least once by 30 September 2021, reflecting referrals to nephrologists. Then, we calculated the frequency of referrals among individuals who met the criteria recommended by the guidelines [6,21,22].

## 3. Results

### 3.1. Clinical Departments Visited by CKD Patients

In September 2021, the total number of outpatients aged ≥18 years was 75,366 (median age, 60 years; males, 52.4%). Furthermore, 12,921 eGFR measurements were performed, of which 3918 (30.3%) had an eGFR < 60 mL/min/1.73 m^2^; and 7770 dipstick tests were performed, of which 1474 (19.0%) had a U-pro result ≥ +1 (Table 1).

Table 2 shows the number of tests for CKD performed in each clinical department by CKD category, reflecting the departments visited by CKD patients. The total number of measurements with an eGFR < 60 mL/min/1.73 m^2^ or a U-pro result ≥ +1 was 4449. Of them, 3065 (68.9%) were performed by any division of internal medicine, not just those departments that are well-known as associated with CKD, meaning that 1384 (31.1%) of tests for CKD were performed by departments other than internal medicine.

#### 3.1.1. Frequency of Testing for Proteinuria or Albuminuria

Table 2 shows the number of CKD patients who were tested for proteinuria or albuminuria at least once within 1 year by CKD category. In September 2020, 15,945 eGFR measurements were performed, of which an eGFR < 60 mL/min/1.73 m^2^ was observed in 5351 (33.6%); and 7377 dipstick tests were performed, of which 1368 (19.0%) had a U-pro result ≥ +1. The total number of CKD patients with an eGFR < 60 or a U-pro result ≥ +1 was 5331. Of them, 3625 (68.0%) underwent a qualitative urine dipstick test, and 1796 (33.7%) underwent a quantitative test (PCR or ACR). All individuals with an eGFR ≥ 60 and a U-pro result ≥ +1 had undergone a dipstick test when CKD was identified.

#### 3.1.2. Frequency of Referral to Nephrologists

Of the 69,071 outpatients at our hospital aged ≥18 years in September 2020 (median age, 60 years; males, 52.9%), 5331 were identified as CKD patients, and 4283 were identified by tests performed in non-nephrology departments at least once in September 2020. We analyzed the number of these patients who visited the nephrology department at least once within 1 year of identification (Table 3). Of them, 27.5% of individuals with an eGFR < 30 mL/min/1.73 m^2^ or U-pro ≥ +1 visited a nephrologist, as recommended by the Kidney Disease: Improving Global Outcomes (KDIGO) guideline [21]. Additionally, 19.7% met the referral criteria recommended by the current Japanese guideline [6,22].

## 4. Discussion

We reviewed electronic medical records of outpatients at a university hospital in central Tokyo, Japan. Our findings revealed that CKD patients visited various clinical departments, and over 30% of them visited departments other than those falling under internal medicine (Table 2). We also analyzed the frequency of proteinuria testing (Table 3) and referral to nephrologists (Table 4) for CKD patients as Qis for CKD care. Of the known Qis, we used those that are expected to be practiced by a wide range of healthcare providers in hospitals [31].

The results presented in Table 2 show that hospitals employ a wide range of healthcare providers who are expected to have a sufficient level of CKD awareness to contribute to the improved care of CKD patients. Thus, the target audience for CKD awareness campaigns needs to be broad. According to a previous study using nationwide hospital-based data in Japan [32], 49.5% of eGFR-defined CKD G3–G5 received treatment from departments other than internal medicine. This result also indicates that CKD patients visit various departments in the hospital, similar to that of this study, in which 31.6% of the tests with the result of eGFR-defined CKD G3–G5 were performed in departments other than internal medicine. In the previous study, the specialties were divided into five categories: cardiology, diabetology, nephrology, internal medicine, and others. However, we collected data from all clinical departments because we are planning to carry out CKD campaigns for each department.

Our findings do not show the degree of CKD awareness in each clinical department. For example, if proteinuria in an individual with an eGFR ≥ 60 mL/min/1.73 m^2^ is reduced to <+1 with proper intervention, they will not be identified as a CKD individual in our study. Of the 69,071 outpatients at our hospital aged ≥18 years in September 2020, 5331 (7.7%) met the CKD criteria. This is lower than the estimated prevalence of CKD that was previously reported in the general Japanese population [33], suggesting the presence of many latent, untested CKD patients [34]. There may also have been cases of CKD that were unrecognized despite testing [35].

Proteinuria is a significant risk factor not only for end-stage renal disease, but also for CVD and all-cause mortality, making it an important target for effective intervention. Therefore, the CKD awareness we consider most important is a further understanding of proteinuria. However, at our hospital, the frequency of dipstick test performance was not optimal, and that of quantitative tests was even lower (Table 3). Dipstick proteinuria is an essential item for annual health checkups provided collectively by insurers/municipalities or privately in Japan [4,36]. Thus, if we have access to the history of these checkup programs, hospital testing is unnecessary—but for now, this is not possible. Furthermore, the frequency of ACR measurements recommended by the KDIGO guidelines [21] was lower than that of PCR measurements. This may be due, in part, to the fact that the ACR test is only reimbursed for the early stages of diabetic nephropathy in Japan.

The sensitivity of dipstick tests is insufficient to detect an ACR >30 mg/gCr or a PCR >150 mg/gCr (stage A2). However, an ACR >300 mg/gCr or a PCR >500 mg/gCr (stage A3), which are a higher risk for CKD and should not be overlooked, can be detected relatively accurately [37,38,39,40,41]. Additionally, in the current Japanese guideline [6,22], U-pro ≥ +1 is considered equivalent to stage A3, at which time referrals are recommended. In Table 4, if U-pro ≥ +1 is regarded as stage A3, 51.5% of G4–G5, 20.2% of A3, and 27.5% of G4–G5 and A3 were referred, as recommended by the KDIGO guideline [21]. Although this result is suboptimal, one reason may be that Table 4 only shows the number of outpatient referrals. It is estimated that there were more referrals for in-patients to nephrologists or other related specialists. The Japanese government recommends that the compliance rate of referral criteria be one of the evaluation indicators for the progress management of CKD measures [6]. However, it is difficult to perform this analysis on a large scale in Japan, so it may be convenient to replace it with the analysis shown in Table 4. Repeated assessments of these QIs, including the use of medications, which can also be extracted from electronic medical records, may be useful for progress management in improving CKD care [42,43]. Thus, research on the preventive effect of these QIs on CKD progression is necessary.

For raising CKD awareness, we plan to inform other departments about the need for proteinuria testing for individuals at high risk of CKD, and the need for early intervention for proteinuria. Additionally, we think it would be effective to conduct an actual condition survey of CKD care in each department for which consent was obtained. Since it is impossible to expect all healthcare providers to diagnose CKD and acquire treatment/care, we would like to inform them about the essentials of CKD care, such as referral to specialists and blood pressure control using RAS inhibitors, according to the characteristics of each department.

Regarding an in-hospital strategy to raise CKD awareness, it is essential to collaborate with medical staff who work outside clinical departments, and have the opportunity to provide early intervention for CKD patients [44]. In Japan, the number of certified kidney disease educators who are qualified nurses/public health nurses, dietitians, and pharmacists is steadily increasing. Additionally, as a collaboration with each clinical department, we believe that sharing information on CKD care according to the characteristics of disease and medication would help raise CKD awareness. The strength of these in-hospital strategies is to facilitate referrals to specialists because CKD patients do not have to visit another hospital for referrals. Additionally, a large number of medical staff work in hospitals.

There are several limitations to our study. First, this investigation was performed at a single institution in Japan. The results are greatly affected by the medical system of each country, and the characteristics or situation of each hospital. For example, there is no obligation to register a primary care physician in Japan, so patients in various situations visit general hospitals. Therefore, it may be difficult to compare the QI values of related studies [17]. Thus, we propose the use of these QIs for progress management in raising CKD awareness in each facility or department for which consent has been obtained. Second, unlike the guideline’s definition [21,22], a single measurement for eGFR and/or U-pro by dipstick test without confirmation of chronicity is used to identify CKD patients, which leads to overdiagnosis [33,45], but, if the analysis is simplified, it may help to track the progress made in improving CKD care by awareness campaigns within the facility. Third, this was a short-term analysis. Although Table 2 shows the results for September 2021, an analysis of September 2020 revealed that there were 5382 CKD cases. Of these tests, 69.1% were performed by any division of internal medicine, which was similar to the results obtained for September 2021 (Table 2). Fourth, our study was performed under COVID-19 pandemic conditions, which might not accurately represent the analysis in a normal situation.

In conclusion, CKD patients visited various clinical departments at our hospital. Therefore, campaigns to raise CKD awareness must reach a wide range of healthcare providers in hospitals. Repeated assessments of the QIs (frequency of proteinuria testing and referral to nephrologists) may be useful for progress management in improving care for CKD patients.

## Figures and Tables

**Table 1:** Descriptive statistics of the study population.

Characteristic	CKD Identification in September 2021 Used for Method 2.1	CKD Identification in September 2020 Used for Method 2.2
Total patients (≥18 years)	75,366	69,071
Median age (years)	60	60
Sex (% male)	52.4%	52.9%
Number of tests for eGFR Results with eGFR < 60	12,921 3918 (30.3%)	15,945 5351 (33.6%)
Number of tests for U-pro Results with U-pro ≥ +1	7770 1474 (19.0%)	7377 1368 (19.0%)
Number of CKD patients (eGFR < 60 or U-pro ≥ +1)	N/A	5331
Follow-up period	N/A	1 year (Until 30 September 2021)

CKD, chronic kidney disease; eGFR, estimated glomerular filtration rate (mL/min/1.73 m^2^); N/A, not applicable.

**Table 2:** The number of tests for CKD performed in each clinical department by CKD category.

Clinical Department	Number of Tests for CKD by Category						
	Total Tests for CKD	G1–G2 eGFR ≥ 60 and U-pro ≥ +1	G3–G5 eGFR <60	G3a eGFR 45–59	G3b eGFR 30–44	G4 eGFR 15–29	G5 eGFR < 15
Overall	4449	531	3918	2401	913	350	254
Internal Medicine	3065	387	2678	1572	645	270	191
Nephrology and Hypertension	928	132	796	295	208	155	138
Diabetes, Metabolism, and Endocrinology	580	145	435	279	112	32	12
Cardiology	408	6	402	244	103	40	15
Clinical Oncology and Hematology	395	12	383	259	94	18	12
Gastroenterology and Hepatology	344	42	302	228	55	10	9
Rheumatology	202	35	167	122	33	10	2
Respiratory Medicine	100	6	94	68	20	3	3
Neurology	56	3	53	40	11	2	0
General Medicine	52	6	46	37	9	0	0
Surgery	396	24	372	254	75	19	24
Urology	348	39	309	188	87	27	7
Obstetrics and Gynecology	107	13	94	61	20	10	3
Emergency Medicine	90	17	73	31	19	9	14
Otorhinolaryngology	73	6	67	59	6	0	2
Dermatology	73	15	58	32	16	6	4
Orthopedic Surgery	71	4	67	49	13	1	4
Cardiac Surgery	46	1	45	26	14	5	0
Neurosurgery	45	12	33	26	6	0	1
Infectious Diseases and Infection Control	37	3	34	33	1	0	0
Ophthalmology	34	0	34	24	4	2	4
Radiology	22	3	19	16	3	0	0
Psychiatry	10	0	10	9	0	1	0
Other departments	32	7	25	21	4	0	0

CKD, chronic kidney disease; eGFR, estimated glomerular filtration rate (mL/min/1.73 m^2^). We calculated the eGFR using an equation designed for the Japanese population, as follows: eGFR = 194 × (sCr) − 1.094 × (age) − 0.287 (×0.739, if female), where sCr = serum creatinine (mg/dL). The sum of the number of CKD tests for each internal medicine division equals the total number of CKD tests performed in the internal medicine department. The number of CKD tests that were classified as G3a, G3b, G4, and G5 equals the total number of tests with G3–G5. If fewer than nine tests for CKD were performed in a department, that department fell into the category of “other departments”.

**Table 3:** Frequency of testing for proteinuria or albuminuria within 1 year of CKD identification.

Number of CKD Patients	Number of CKD Patients Who Had Each Test and Its Frequency			
CKD category	Dipstick test	PCR	ACR	PCR or ACR
eGFR < 60 or U-pro ≥ +1 (*n* = 5331)	3625 (68.0%)	1431 (26.8%)	553 (10.4%)	1796 (33.7%)
G1-G2: eGFR ≥ 60 and U-pro ≥ +1 (*n* = 451)	451 (100%)	170 (37.7%)	87 (19.3%)	231 (51.2%)
G3a: eGFR 45–59 (*n* = 3017)	1884 (62.4%)	490 (16.2%)	289 (9.6%)	703 (23.3%)
G3b: eGFR 30–44 (*n* = 1144)	836 (73.1%)	364 (31.8%)	130 (11.4%)	444 (38.8%)
G4: eGFR 15–29 (*n* = 417)	364 (87.3%)	250 (60.0%)	39 (9.4%)	260 (62.4%)
G5: eGFR < 15 (*n* = 302)	90 (70.2%)	157 (52.0%)	8 (2.6%)	158 (52.3%)

CKD, chronic kidney disease; eGFR, estimated glomerular filtration rate (mL/min/1.73 m^2^); PCR, urine protein-to-creatinine ratio; ACR, urine albumin-to-creatinine ratio.

**Table 4:** Frequency of referral to nephrologists within 1 year of CKD identification.

	Number of CKD Patients Who Visited the Nephrology Department and Its Frequency			
CKD category	U-pro (−) or no test	U-pro (±)	U-pro ≥ +1	Total
G1: eGFR ≥ 90	N/A		26/353 (7.4%)	26/353 (7.4%)
G2: eGFR 60–89	N/A			
G3a: eGFR 45–59	N/A	15/198 (7.6%)	32/176 (18.2%)	47/374 (12.6%)
G3b: eGFR 30–44	97/772 (12.6%)		37/127 (29.1%)	134/899 (14.9%)
G4: eGFR 15–29	68/172 (39.5%)		35/64 (54.7%)	103/236 (43.6%)
G5: eGFR < 15	60/97 (61.9%)		20/22 (90.9%)	80/119 (67.2%)
Total	240/1239 (19.4%)		150/742 (20.2%)	390/1981 (19.7%)

CKD, chronic kidney disease; eGFR, estimated glomerular filtration rate (mL/min/1.73 m^2^); N/A, not applicable.
